# Comparative Analysis of Short- and Long-Term Surgical Outcomes Following Open Conversion from Laparoscopic and Robotic Gastrectomy in Patients with Gastric Cancer

**DOI:** 10.3390/jcm15156036

**Published:** 2026-08-03

**Authors:** Mahmoud Hemdan, Dong-eun Lee, Sang Soo Eom, Young Shick Rhee, Woo Jun Ahn, Bang Wool Eom, Young-Woo Kim, Hong Man Yoon, Keun Won Ryu

**Affiliations:** 1 Center for Gastric Cancer, National Cancer Center, Goyang 10408, Republic of Korea; m.abouellail24@gmail.com (M.H.); awj89@ncc.re.kr (W.J.A.); kneeling79@ncc.re.kr (B.W.E.); youngwookim@ncc.re.kr (Y.-W.K.); 2 Surgical Oncology Department, Maadi Military Medical Complex, Cairo 4211220, Egypt; 3 Biostatistics Collaboration Team, Research Institute, National Cancer Center, Goyang 10408, Republic of Korea; 4 Department of Surgery, Inje University College of Medicine, Ilsan Paik Hospital, Goyang 10408, Republic of Korea; 5 Department of Surgery, Seoul National University College of Medicine, Seoul 03080, Republic of Korea

**Keywords:** gastric cancer, open conversion, laparoscopic gastrectomy, robotic gastrectomy, short-term complication, long-term survival

## Abstract

**Background/Objective**: Laparoscopic gastrectomy (LG) and robotic gastrectomy (RG) exhibit similar surgical outcomes in patients with gastric cancer (GC), except for the higher cost associated with RG. Comparative surgical outcome studies after open conversion from laparoscopic (OL) and robotic (OR) gastrectomy in GC are limited. This study aimed to evaluate clinical effectiveness while considering differences in procedural cost. **Methods**: This retrospective cohort study included a comparative analysis of patients with GC who underwent OL or OR. Inclusion criteria were histologically confirmed adenocarcinoma and curative resection for GC using OL or OR. Exclusion criteria included initially planned open surgery and other laparoscopic or robotic procedures without gastrectomy. Clinicopathological characteristics, as well as short- and long-term surgical outcomes, were compared between groups. **Results**: Overall, 118 (1.9%) of 6336 LG cases and 10 (2.6%) of 384 RG cases were included (*p* = 0.3308). Clinicopathological characteristics were statistically comparable between groups. Postoperative complication incidence, evaluated using the Clavien–Dindo classification, was 19.5% and 10% in the OL and OR groups, respectively (*p* > 0.05). The median hospital stay was 8 and 9.5 days in the OL and OR groups, respectively (*p* > 0.05). Reasons for open conversion were similar between groups and included adhesion, bleeding, advanced disease, and positive resection margins, among others. Overall 5-year survival was 0.79 (0.72–0.88) and 0.88 (0.67–1.0) in OL and OR, respectively (*p* = 0.596). **Conclusions**: Short- and long-term surgical outcomes after OL and OR did not differ statistically. Selection of LG versus RG should be performed with caution.

## 1. Introduction

Gastric cancer (GC) is the 5th most commonly diagnosed malignancy worldwide and ranks 4th for cancer-related mortality. Nonetheless, its global incidence has declined in recent decades [1]. The age-standardized incidence rate per 100,000 individuals remains highest in East Asia, including South Korea, in both sexes [2]. Early detection through national and public screening programs, together with advances in treatment, has increased the proportion of surgically treated early gastric cancer (EGC) cases from 28.6% in 1995 to 63.6% in 2019; the 5-year survival rate in South Korea also improved from 43.9% (1993–1995) to 77.5% (2015–2019) [2].

Minimally invasive surgery (MIS), using laparoscopic or robotic approaches, has become the standard of care for curative intent treatment of GC at many high-volume centers, particularly in East Asia [3]. Laparoscopic gastrectomy (LG) is widely accepted for GC. However, the role of robotic gastrectomy (RG) remains insufficiently investigated [4]. Intraoperative safety risks and constraints in applying appropriate oncologic techniques may necessitate open conversion (OC) from LG or RG, raising concerns regarding suboptimal surgical outcomes [5,6]. Several studies have reported variable surgical outcomes after OC from LG (OL). Nonetheless, studies of OC from RG (OR) remain limited, including those evaluating surgical outcomes. Given the higher cost associated with OR, comparative studies of OL and OR are warranted [4].

This study aimed to compare clinical outcomes after OL and OR in patients with GC and to evaluate short-term surgical and long-term survival outcomes in the MIS era.

## 2. Materials and Methods

### 2.1. Collection of Surgical Data

This cohort study was a retrospective analysis of a prospectively established database, including LG for GC performed at the National Cancer Center, Korea, between 1 January 2003, and 30 April 2025, and RG for GC performed between 1 February 2009, and 30 April 2025. The National Cancer Center, Korea, is a high-volume cancer center with a structured database and accurate long-term follow-up. LG for GC has been performed since 2002, and RG for GC since 2009, at the National Cancer Center, Korea.

The inclusion criteria were histologically confirmed GC, planned curative resection by LG or RG, and follow-up of at least 3 months. The exclusion criteria included planned open surgery at the outset, other laparoscopic or robotic procedures without gastrectomy, and cases lacking clear OC data.

Collected data included age, sex, body mass index (BMI), previous abdominal surgery, previous gastric surgery, adjuvant chemotherapy, and preoperative status assessed using the American Society of Anesthesiologists (ASA) score [7]. Operative data included gastrectomy type, reconstruction, lymph node dissection, number of harvested lymph nodes, and lengths of the resected distal and proximal margins. The reasons for intraoperative OC in LG and RG were also collected and compared.

Pathological data included tumor location and size, histologic differentiation, T classification, number of harvested lymph nodes, N classification, and final staging based on the American Joint Committee on Cancer (AJCC) 8th edition [8]. Postoperative short-term outcomes included complications graded using the Clavien–Dindo (C-D) classification and hospital stay (days) [9]. Long-term overall 5-year survival was also compared between the OL and OR groups.

This study was approved by the Institutional Review Board of the National Cancer Center (No. NCC2025-0364), which waived the need for informed consent because of the retrospective design.

### 2.2. Statistical Analysis

Continuous variables are presented as medians (range) and were compared using the Wilcoxon rank sum test. Categorical variables are expressed as numbers and percentages and were compared using the chi-square test or Fisher’s exact test, as appropriate. Overall survival (OS) was defined as the time from the date of surgery to death from any cause or last follow-up. Survival curves were estimated using the Kaplan–Meier method and compared between groups using the log-rank test. Restricted mean survival time (RMST) was additionally calculated up to 60 months (τ = 60 months) to provide an intuitive comparison of survival between groups. To identify factors associated with 5-year overall survival, Cox proportional hazards regression analysis was performed. A two-sided (*p* < 0.05) was considered statistically significant, and analyses were performed using R version 4.5.2 (R Foundation for Statistical Computing, Vienna, Austria).

## 3. Results

### 3.1. Characteristics of the Study Population

**Figure 1:**
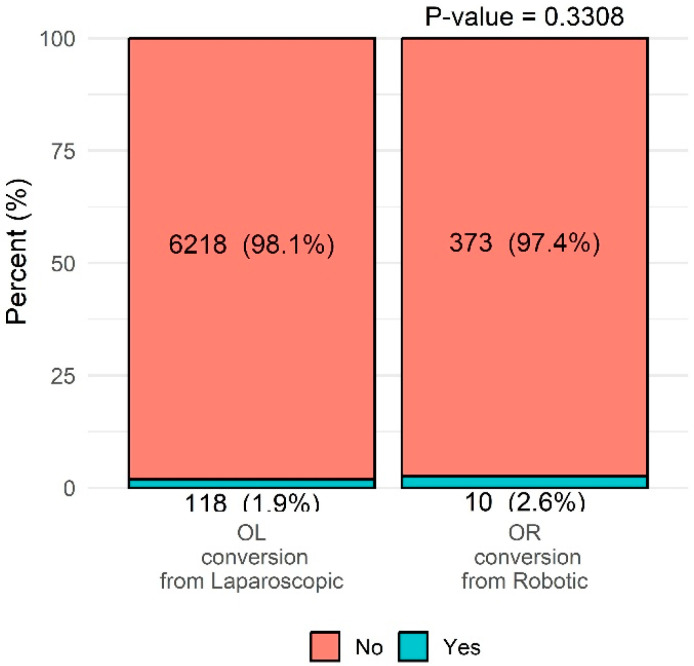
Comparison of total cases between OL and OR.

During the study period, 118 (1.9%) of the 6336 LG and 10 (2.6%) of the 384 RG cases were included (*p* = 0.3308) (Figure 1).

Clinical factors, including age, sex, BMI, previous surgical history, and ASA score, did not differ significantly between the OL and OR groups. (*p* > 0.05) (Table 1).

Pathological stage and other tumor characteristics did not differ significantly between the OL and OR groups (*p* > 0.05). Most cases in both groups were early-stage disease, and the adjuvant chemotherapy rate was not significantly different between groups.

### 3.2. Surgical Procedures and Outcomes

The type of gastrectomy, reconstruction method, and extent of lymph node dissection did not differ significantly between the OL and OR groups (*p* > 0.05), respectively (Table 2).

The number of harvested lymph nodes and resected margins were also not significantly different (*p* > 0.05).

Postoperative complications classified by C-D occurred in 23 patients (19.5%) in the OL group and one patient (10%) in the OR group, with no significant between-group difference (*p* > 0.05) in the OL group, one patient (Grade V) died of asphyxia, and one patient (Grade IIIb) experienced an esophagojejunal anastomosis leakage that was managed with stent insertion and percutaneous transhepatic biliary drainage for peribiliary metastasis. The median postoperative hospital stay was 8 (range, 3–120) and 9.5 (range, 7–36) days in the OL and OR groups, respectively (*p* = 0.3918).

### 3.3. Causes of Open Conversion and Chronological Distribution of OL and OR

**Figure 2:**
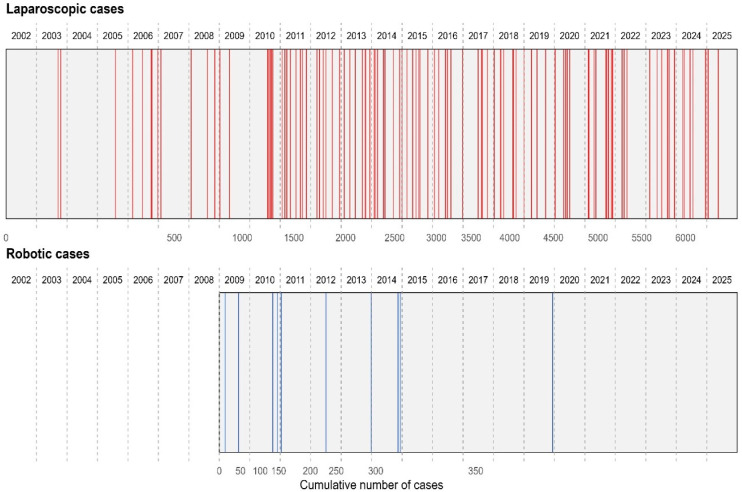
Chronological distribution of OL and OR from the whole LG and RG series.

The most common cause of OL was adhesions (28.8%), followed by advanced disease (28%), bleeding (22.9%), positive resection margins (6.8%), and anastomotic difficulty (6.8%) (Table 3). In contrast, bleeding (40%) was the leading cause of OR, followed by adhesions (30%), advanced disease (20%), and positive resection margins (20%); overall, the causes of OC were similar between the two groups (*p* > 0.05). The chronological distribution as shown in (Figure 2) open conversions in the laparoscopic series (OL) were distributed relatively evenly over the study period, whereas conversions in the robotic series (OR) were clustered predominantly during the early phase of the robotic program.

### 3.4. Long-Term Survival and Multivariate Analysis of Survival

**Figure 3:**
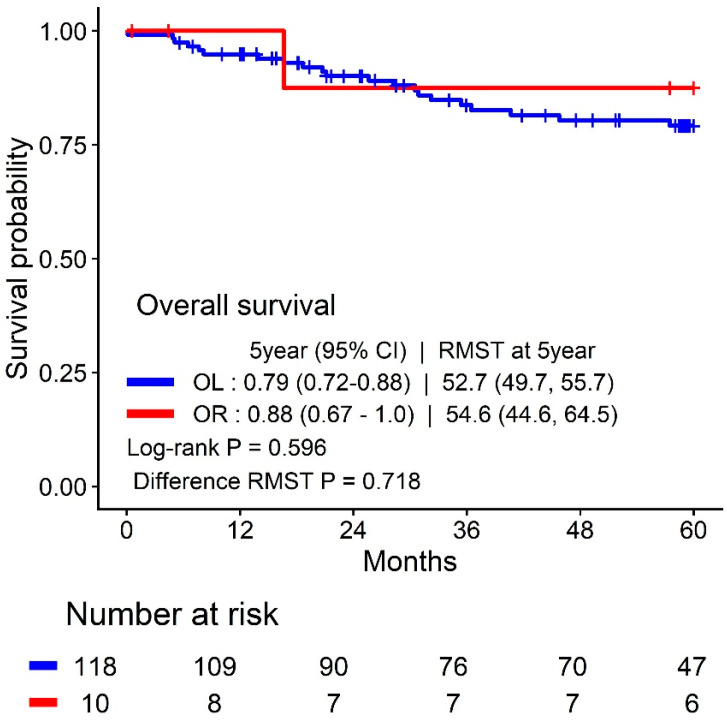
Kaplan–Meier curves comparing 5-year survival between groups. CI: confidence interval; OL: open conversion from laparoscopic surgery; OR: open conversion from robotic surgery; RMST: restricted mean survival time.

The median follow-up duration for the overall cohort was 70.4 months (95% confidence interval (CI), 59.3–85.0). The Kaplan–Meier survival curves are displayed in Figure 3. The RMST at 60 months was 52.7 months (95% CI, 49.7–55.7) and 54.6 months (95% CI, 44.6–64.5) in OL and OR, respectively, corresponding to an approximately 1.9-month longer RMST for OR, although the difference was not statistically significant. In the univariate analysis, pathological stage was the only factor significantly associated with overall survival. In the multivariable Cox proportional hazards model adjusted for pathological stage, surgery type was not significantly associated with overall survival (*p* = 0.627, Table 4).

## 4. Discussion

Minimally invasive surgery (MIS) has become widely adopted, and surgeons increasingly seek safe, less invasive procedures to avoid open surgery, minimize tissue damage and manipulation, reduce postoperative complications, shorten hospital stays, and lower overall costs. Many studies comparing the clinical outcomes of LG and RG for GC have reported no differences in short- and long-term outcomes, with less blood loss but longer operative time in RG [10]. OC retains the drawbacks of open surgery, whereas MIS is associated with higher costs, and clinical outcomes remain a concern. This study may be among the first to compare OL and OR for GC with respect to short-term postoperative complications and long-term survival; no clinically relevant differences were observed between groups.

Several studies have reported an OL incidence ranging from 0% to 17.4%, with bleeding, adhesions, and advanced tumors being the most common causes [5]. In this study, OL and OR rates were 1.9% and 2.6%, respectively, which were low compared with the 7.8% reported in other studies, possibly because the procedures were performed at a qualified, high-volume center [11].

Compared with OG, LG was associated with longer operative time, less intraoperative blood loss, and faster postoperative recovery. The number of harvested lymph nodes, in-hospital mortality, and tumor recurrence rates were similar between the groups. Postoperative morbidity and 5-year overall survival favored LG, although the cost of LG was 3.0% higher than that of OG [12,13]. Nevertheless, the use of robotic systems remains controversial, mainly because of their substantial expense relative to other procedures. A retrospective analysis investigated differences in overall costs between robotic and laparoscopic surgery [14]. In that report, higher cost was identified as the main disadvantage. The main drivers of the higher cost associated with robotic gastrectomy are the cost of the robotic system itself and the depreciation reserve. As other reports have indicated, the current high costs are not widely acceptable worldwide.

Studies from developed countries have demonstrated that RG with systematic lymph node dissection may be safe, with short- and long-term oncologic outcomes equivalent to those of either LG or OG for the surgical treatment of GC. However, the high cost remains the primary barrier to justifying RG as a routine, standard treatment for patients with gastric cancer in developing or underdeveloped countries [15,16]. At the National Cancer Center (NCC), Korea, LG began in 2002, and most surgeons are thought to have overcome the learning curve. LG was initially indicated for EGC; as clinical evidence supporting LG in advanced gastric cancer (AGC) accumulated, LG was gradually extended to AGC, and OL became more evenly distributed over time as the indication expanded chronologically [17,18,19]. Nonetheless, RG began in 2009 at the NCC, and its indication remains limited to EGC because of limited evidence for its use in AGC. Therefore, OR cases were predominantly observed during the early period, likely reflecting the learning curve.

This study had some limitations. First, the number of OR cases was small compared with OL cases, which limited the statistical analysis of clinical outcomes and precluded definitive evidence. Second, because this was a retrospective database analysis, the chronology and indications differed between LG and RG. Accordingly, multicenter analyses are warranted to evaluate the cost–benefit of RG for OR with respect to clinical outcomes and current cost difference.

## 5. Conclusions

Short-term postoperative complications, hospital length of stay, and long-term survival after OL and OR did not differ significantly. Selection between LG and RG should be approached cautiously. Further multicenter studies in larger populations are warranted.

## Figures and Tables

**Table 1:** Characteristics of the study population.

Variables		OL	OR	*p*-Value
		N = 118	N = 10	
Age				0.9398 †
	Median (Min–Max)	65.0 (28.0–87.0)	64.0 (53.0–84.0)	
Sex				0.2887 ‡
	Male	82 (69.5%)	5 (50.0%)	
	Female	36 (30.5%)	5 (50.0%)	
BMI				0.3940 †
	Median (Min–Max)	23.8 (15.9–35.3)	25.2 (19.2–30.1)	
Previous abdominal surgery				>0.9999 ‡
	No	86 (72.9%)	8 (80.0%)	
	Yes	32 (27.1%)	2 (20.0%)	
Previous gastric surgery				>0.9999 ‡
	No	109 (92.4%)	10 (100.0%)	
	Yes	9 (7.6%)	0 (0.0%)	
ASA score				0.4149 ‡
	1	32 (27.1%)	2 (20.0%)	
	2	68 (57.6%)	8 (80.0%)	
	3	18 (15.3%)	0 (0.0%)	
T classification				0.6192 ‡
	T1	65 (55.1%)	7 (70.0%)	
	T2	15 (12.7%)	1 (10.0%)	
	T3	17 (14.4%)	2 (20.0%)	
	T4	20 (16.9%)	0 (0.0%)	
	ypT0	1 (0.8%)	0 (0.0%)	
Location				0.9308 ‡
	upper 1/3	37 (31.4%)	3 (30.0%)	
	middle 1/3	42 (35.6%)	3 (30.0%)	
	lower 1/3	37 (31.4%)	4 (40.0%)	
	whole stomach	2 (1.7%)	0 (0.0%)	
Size longest diameter				0.3090 †
	Median (Min–Max)	3.6 (0.6–18.9)	6.1 (1.6–8.9)	
Differentiation				0.8125 ‡
	Dif	45 (38.1%)	3 (30.0%)	
	Undif	69 (58.5%)	7 (70.0%)	
	no residual	4 (3.4%)	0 (0.0%)	
N classification				0.7908 ‡
	N0	87 (73.7%)	7 (70%)	
	N1	9 (7.6%)	1 (10%)	
	N2	11 (9.3%)	1 (10%)	
	N3a	4 (3.4%)	0 (0%)	
	N3b	7 (5.9%)	1 (10%)	
Pathological stage				0.6916 ‡
	yp 0	1 (0.8%)	0 (0.0%)	
	I	74 (62.7%)	8 (80.0%)	
	II	19 (16.1%)	0 (0.0%)	
	III	20 (16.9%)	2 (20.0%)	
	IV	4 (3.4%)	0 (0.0%)	
Adjuvant chemotherapy				0.7222 ‡
	No	82 (69.5%)	8 (80.0%)	
	Yes	36 (30.5%)	2 (20.0%)	

OL: open conversion from laparoscopic surgery; OR: open conversion from robotic surgery; †: Wilcoxon rank sum test; ‡: Fisher’s exact test; ASA: American Society of Anesthesiologists. Pathological staging was according to the AJCC 8th edition.

**Table 2:** Surgical procedures and outcomes.

Variables		OL	OR	*p*-Value
		N = 118	N = 10	
Surgical procedures				
Type of gastrectomy				0.8712 ‡
	Distal	62 (52.5%)	5 (50%)	
	Total	48 (40.7%)	5 (50%)	
	Proximal	7 (5.9%)	0 (0%)	
	PPG	1 (0.8%)	0 (0%)	
Reconstruction				>0.9999 ‡
	Billroth I	19 (16.1%)	2 (20%)	
	Billroth II	39 (33.1%)	3 (30%)	
	Roux-en-Y	52 (44.1%)	5 (50%)	
	Double tract method (EJ + GJ + JJ)	4 (3.4%)	0 (0%)	
	Esophagogastrostomy	3 (2.5%)	0 (0%)	
	Gastrogastrostomy	1 (0.8%)	0 (0%)	
Lymph node dissection				0.7222 ‡
	Less than D2	36 (30.5%)	2 (20%)	
	D 2 or more	82 (69.5%)	8 (80%)	
Harvest LNs				
	Median (min–max)	34 (2–91)	35 (24–55)	0.4996 †
Length of resection margin				
Proximal				
	N	117	10	
	Median (min–max)	3.6 (0.5–17.5)	3.4 (0.5–5.5)	0.3703 †
Distal				
	N	117	10	
	Median (min–max)	6 (0–18)	8 (3–19.7)	0.0792 †
Postoperative data				
Complication grade *				0.4792 ‡
	No complication	95 (80.5%)	9 (90%)	
	I	6 (5.1%)	0 (0%)	
	II	4 (3.4%)	1 (10%)	
	IIIa	11 (9.3%)	0 (0%)	
	IIIb	1 (0.8%)	0 (0%)	
	V	1 (0.8%)	0 (0%)	
Hospital stay (days)				0.3918 †
	Median (Min–Max)	8 (3–120)	9.5 (7–36)	

OL: open conversion from laparoscopic surgery, OR: open conversion from robotic surgery, †: Wilcoxon rank sum test, ‡: Fisher’s exact test, *: Clavien–Dindo classification.

**Table 3:** Causes of open conversion.

Variables	OL	OR	*p*-Value ‡
	N = 118	N = 10	
Adhesion	34 (28.8%)	3 (30%)	>0.9999
Bleeding	27 (22.9%)	4 (40%)	0.254
Advanced disease	33 (28%)	2 (20%)	0.727
Positive margin	8 (6.8%)	2 (20%)	0.176
anastomosis difficulty	8 (6.8%)	0 (0%)	>0.9999
Co2 related	2 (1.7%)	0 (0%)	>0.9999
Huge abdominal cavity	2 (1.7%)	0 (0%)	>0.9999
Small abdominal cavity	0 (0%)	1 (10%)	0.0781
Colonic mesentery large defect	1 (0.8%)	0 (0%)	>0.9999
for omentectomy	1 (0.8%)	0 (0%)	>0.9999
Unknown	2 (1.7%)	0 (0%)	>0.9999

OL: open conversion from laparoscopic surgery, OR: open conversion from robotic surgery, ‡: Fisher’s exact test.

**Table 4:** Cox proportional hazards model for overall 5-year survival.

Variables		N	Event	Univariable		Multivariable	
				HR (95% CI)	*p*-Value	HR (95% CI)	*p*-Value
Age		128	22	1.015 (0.978–1.052)	0.433		
Sex	Male	87	14	1 (ref)			
	Female	41	8	1.332 (0.558–3.177)	0.518		
BMI		128	22	1.006 (0.892–1.133)	0.926		
Previous abdominal surgery	No	94	15	1 (ref)			
	Yes	34	7	1.59 (0.646–3.914)	0.313		
Previous gastric surgery	No	119	21	1 (ref)			
	Yes	9	1	0.732 (0.098–5.446)	0.761		
ASA score	1~2	110	17	1 (ref)			
	≥3	18	5	2.334 (0.859–6.341)	0.096		
Pathological stage	yp0 + I + II	102	12	1 (ref)		1 (ref)	
	III + IV	26	10	4.462 (1.923–10.36)	<0.001	4.447 (1.9156–10.322)	0.001
Differentiation	Dif	48	6	1 (ref)			
	undif + no residual	80	16	1.668 (0.652–4.263)	0.286		
Operation	Lap. conv.	118	21	1 (ref)		1 (ref)	
	Robot conv.	10	1	0.585 (1.711–4.348)	0.600	0.608 (0.082–4.524)	0.627

BMI: body mass index; CI: confidence interval; HR: hazard ratio.

## Data Availability

The data presented in this study are available on request from the corresponding author due to privacy or ethical restrictions.

## Abbreviations

The following abbreviations are used in this manuscript:

AGC	Advanced gastric cancer
AJCC	American Joint Committee on Cancer
ASA	American Society of Anesthesiologists
BMI	Body mass index
C-D	Clavien–Dindo
CI	Confidence interval
EGC	Early gastric cancer
GC	Gastric cancer
HR	Hazard ratio
LG	Laparoscopic gastrectomy
MIS	Minimally invasive surgery
NCC	National Cancer Center
OC	Open conversion
OL	Open conversion from laparoscopic surgery
OR	Open conversion from robotic surgery
OS	Overall survival
RG	Robotic gastrectomy
RMST	Restricted mean survival time
